# Annexin A4 fucosylation enhances its interaction with the NF-kB p50 and promotes tumor progression of ovarian clear cell carcinoma

**DOI:** 10.18632/oncotarget.10226

**Published:** 2016-06-22

**Authors:** Huimin Wang, Lu Deng, Mingbo Cai, Huiyu Zhuang, Liancheng Zhu, Yingying Hao, Jian Gao, Juanjuan Liu, Xiao Li, Bei Lin

**Affiliations:** ^1^ Department of Obstetrics and Gynecology, Shengjing Hospital Affiliated to China Medical University, Shenyang, Liaoning, China; ^2^ Department of Obstetrics and Gynecology, Hospital of Zhengzhou University, Zhengzhou, Henan, China; ^3^ Department of Obstetrics and Gynecology, Beijing Chaoyang Hospital Affiliated to Capital Medical University, Beijing, China

**Keywords:** ovarian clear cell carcinoma, ANXA4, Lewis y antigen, NF-kB p50, biological behavior

## Abstract

**Objective:**

To study the structural relationship between annexin A4 and the Lewis y antigen and compare their expression and significance in ovarian clear cell carcinoma, and to explore how annexin A4 fucose glycosylation effects the interaction between annexin A4 and NF-kB p50, and how it promotes tumour progression of ovarian clear cell carcinoma.

**Methods:**

Structural relationships between annexin A4 and Lewis y antigen were detected using immunoprecipitation. Annexin A4 and Lewis y antigen expression in various subtypes of ovarian cancer tissues was detected by immunohistochemistry, and the relation between their expression was examined. Any interactions between annexin A4 and NF-kB p50 in ovarian clear cell carcinoma were detected by co-immunoprecipitation. Then looked for changes in expression of Lewis y antigen, annexin A4, NF-kB p50 and a number of downstream related molecules before and after transfection annexin A4 or FUT1, and also analyzed changes in biological processes.

**Results:**

Lewis y antigen is a part of annexin A4 structure. The expression rate of both annexin A4 and Lewis y antigen was significantly higher in ovarian clear cell carcinoma than in other subtypes of epithelial ovarian cancer, and are associated with the clinical stages, chemotherapy resistance and poor prognostic. The interaction between annexin A4 and NF-kB p50 promoted cell proliferation, adhesion, invasion, metastasis ability and autophagy, and inhibits apoptosis, Lewis y enhanced this interaction.

**Conclusion:**

Annexin A4 contains Lewis y structure, Lewis y antigen modification of annexin A4 enhances its interaction with NF-kB p50, which promotes ovarian clear cell carcinoma malignancy progression.

## INTRODUCTION

Ovarian clear cell carcinoma (OCCC) is a rare pathologic subtype of epithelial ovarian cancer (OC), accounting for 5%-25% of epithelial OC, and sometimes arising from endometriosis. OCCC is more malignant than other subtypes of epithelial ovarian cancer, it also exhibits low chemosensitivity to paclitaxel plus platinum-based treatment and greater resistance to chemotherapy. Despite a higher rate of early detection, the five-year survival rate of OCCC is only 27% [1]. At the time of this study, many challenges in the treatment of OCCC remain: OCCC pathogenesis is unclear; known ovarian cancer-related tumor markers such as CA125, HE4, and others have relatively low sensitivity and specificity for OCC; and no ideal treatment regime for OCCC patients exists.

Annexin A4 (ANXA4), or inner fusion protein, is a member of the important annexin family. Recent studies have shown that, ANXA4 can reversibly bind to membrane phospholipids in a calcium-dependent manner, and is involved in regulating tumor cell proliferation, apoptosis, adhesion, invasion, metastasis, and chemotherapy resistance, as well as other biological processes [2–4]. In 2012, Atsuhiko Toyama [5] used cDNA microarray assay to show that ANXA4 is one of several genes over-expressed in OCCC. Immunohistochemistry confirmed that there were elevated ANXA4 levels in OCCC and low ANXA4 levels in other ovarian cancer subtypes, suggesting that ANXA4 can serve as an OCCC-specific marker gene.

Lewis y antigen, a double fucose oligosaccharide, is a terminal oligosaccharide of certain glycoproteins and glycolipids, high Lewis y antigen expression is associated with a various processes in tumor progression. Previous studies have shown a close relationship between Lewis y antigen and certain processes involved in malignancy progression, including adhesion, recognition, malignant transformation of cells, immune escape, invasion and metastasis, and chemotherapy resistance, as well as others [6–10]. Many ovarian cancer marker molecules, such as CA125, CD44, and HE4, contain Lewis y antigen structure, and Lewis y antigen modifications also enhance the processes mediated by these molecules that promote tumor occurrence and development [8, 11].

In the annexin family, core fucose glycosylation has been found on ANXA1 [12]. We have previously shown the presence of Lewis y antigen structure on ANXA2 and that Lewis y modification promoted ANXA2-mediated tumor adhesion, metastasis, and other malignancy progression processes [13]. Currently, the effects of ANXA4 glycosylation modification are unclear, as are its roles in the process of tumor occurrence and development that involve ANXA4.

In this study, immunoprecipitation (IP) and confocal laser scanning microscopy were used to study the structural relationship between ANXA4 and Lewis y antigen. To analyze the relationship between their expression and OCCC clinicopathological parameters and prognosis, ANXA4 and Lewis y antigen expression levels were determined in various subtypes of epithelial ovarian cancer tissues and cells, using, in order, immunohistochemistry (IHC), immunocytochemistry (ICC), Western blots, and real-time PCR. To elucidate the relationship between ANXA4 and NF-kB p50, we examined changes in OCCC cells that were modified to produce high ANXA4 levels (high transfection) or low ANXA4 levels (knockdown ANXA4) in terms of the expression changes of related molecules and malignant processes in cells relative to the unmodified OCCC cells. We also looked for effects of Lewis y antigen modification of ANXA4 that were common to both ANXA4 and NF-kB p50 and analyzed how this modification influenced OCCC occurrence, development, and mechanism, in order to provide a stronger theoretical foundation for OCCC diagnosis and treatment.

## RESULTS

### Co-expression of Lewis y antigen and ANXA4 in OCCC cells

**Figure 1:**
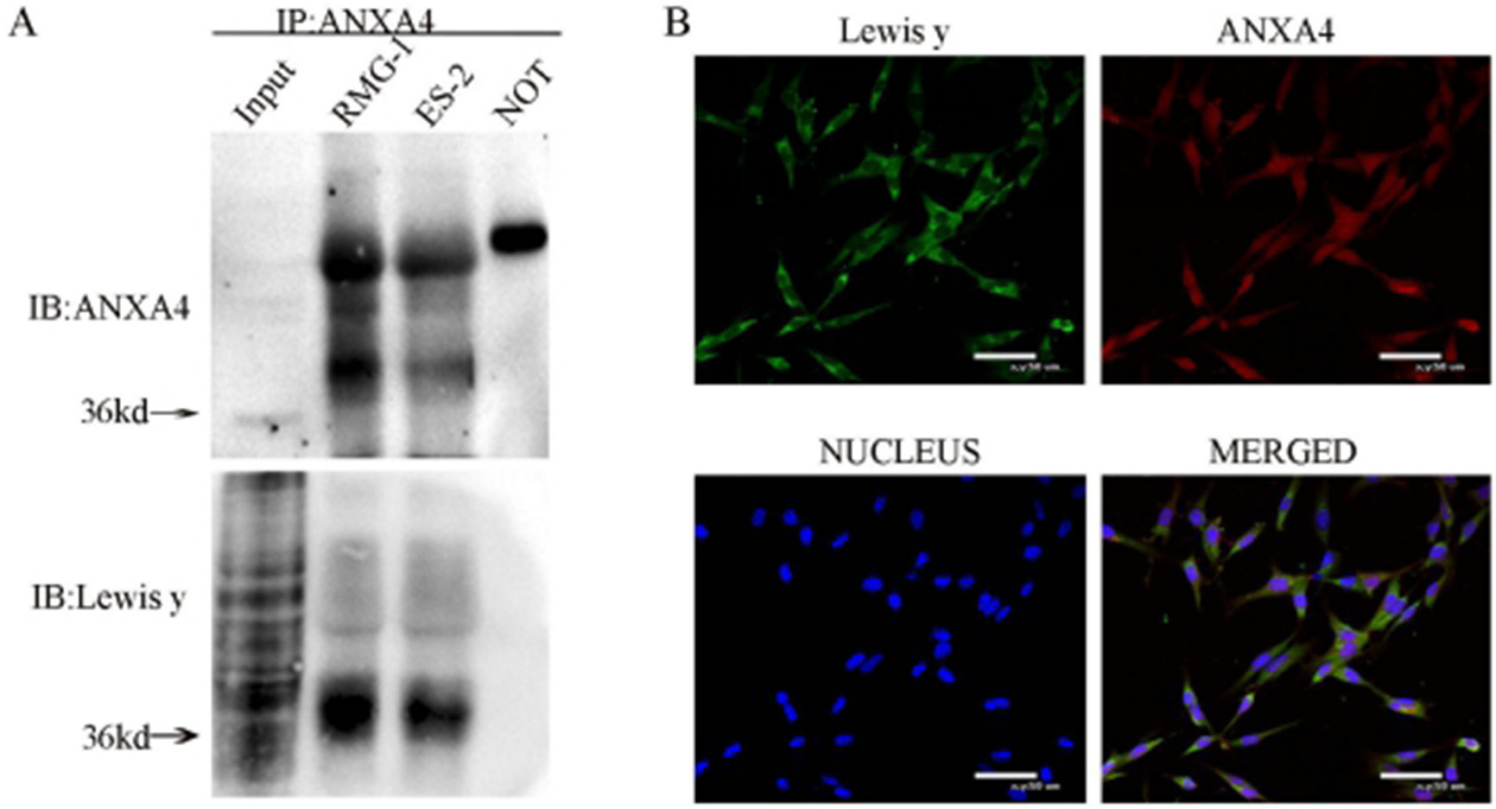
Interaction of ANXA4 and Lewis y in RMG-1 and ES-2 cell lines Cell lystes from RMG-1 and ES-2 were immunoprecipitated with anti-ANXA4 antibody, then immunoblotted with anti-ANXA4 and anti-Lewis y antibodies **(A)** Double-labeling immunofluoscence shows the co-localization of ANXA4 and Lewis y in OCCC carcinoma ES-2 cells **(B)**.

Co-expression of ANXA4 and Lewis y antigen in two OCCC cell lines (RMG-1 and ES-2) was detected using immunoprecipitation. The molecular weight of ANXA4 containing Lewis y antigen structure was found to be about 36 kDa. No expression of ANXA4 or Lewis y antigen was seen in the negative control (Figure 1A).

In the double fluorescence confocal experiment, red fluorescence-labeled ANXA4 was observed to be widely distributed in the cell membrane, cytoplasm and nuclei, the green fluorescence-labeled Lewis y antigen was mainly located in the cell membrane and cytoplasm, with a small amount also seen in the cell nuclei, along with the blue fluorescence of DAPI staining. According to the image analysis software, overlap of red fluorescence-labeled ANXA4 with green fluorescence-labeled Lewis y antigen appears as yellow fluorescence, so co-localization of Lewis y antigen and ANXA4 was demonstrated (Figure 1B).

### Expression of ANXA4 and Lewis y antigen in different subtypes of ovarian cancer tissues and normal ovarian tissues

Positive expression of ANXA4 was widely present in cell membranes, cytoplasm, and nuclei. The ANXA4 positive expression rate in OCCC was 95.3%(82/86), which was significantly higher than in other subtypes of OC 40.0%(22/55) or in normal ovarian tissues( 0%,0/10), all *p* <0.01(*P*=0.000, 0.000).

**Figure 2:**
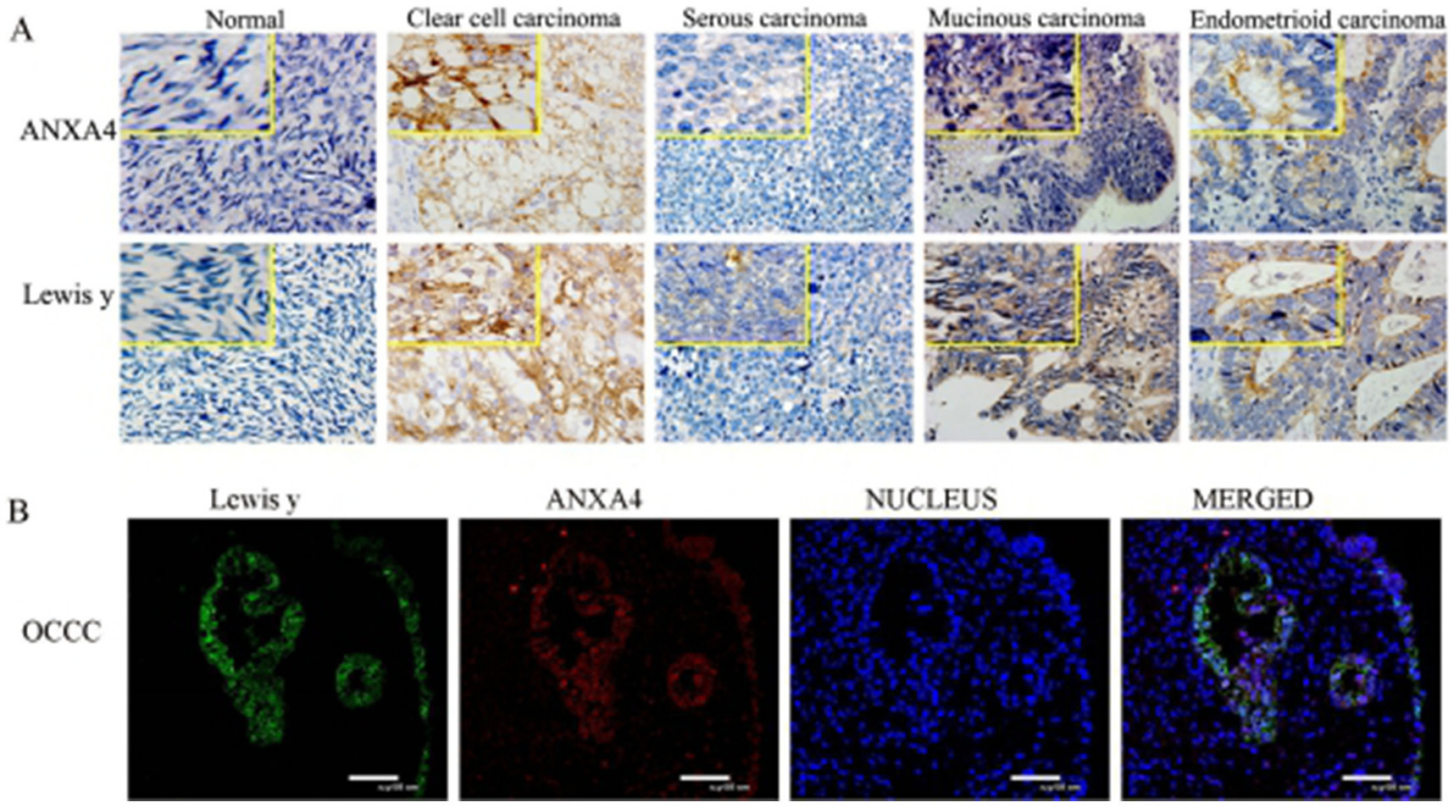
Expression of ANXA4 and Lewis y in various ovarian tissue (400 ^*^) (**(A)** IHC detect the expression in normal ovarian tissue and tissue from various subtypes of OC; **(B)** confocal laser scanning microscopy detect the expression in OCCC tissue).

The positive expression of Lewis y antigen was present mainly in cell membranes and cytoplasm, but the nucleus also show visible coloration, similar to what was observed for ANXA4 expression. The Lewis y antigen positive expression rate in OCCC tissue was 96.5%(83/86), which was significantly higher than in other subtypes of OC (85.5%, 47/55)and normal ovarian tissue (0%, 0/10), all *p* < 0.05 (*P*=0.039, 0.000). (See Table 1 and Figure 2)

**Table 1:** Expression of ANXA4 and Lewis^y^ antigen in ovarian tumors and normal ovarian tissue

Characteristics	Cases	ANXA4						Lewis y					
		-	+	++	+++	Positive cases	Positive rates (%)	-	+	++	+++	Positive cases	Positive rates (%)
**Normal**	10	10	0	0	0	0	0^2^	10	0	0	0	0	0^4^
**OCCC**	86	4	28	30	24	82	95.3^1^	3	28	20	35	83	96.5^3^
**Other subtypes**	55	33	12	6	4	22	40.0	8	18	12	17	47	85.5
**Serous**	30	22	7	0	1	8	26.7	5	8	8	9	25	83.3
**Mucinous**	17	6	3	6	2	11	64.7	3	7	3	4	14	82.4
**Endometrioid**	8	5	2	0	1	3	37.5	0	3	1	4	8	100

Note: ^1, 3^ Comparison of the expression of ANXA4 and Lewis y antigen in OCCC and other OC subtypes

^2, 4^Comparison of the expression of ANXA4 and Lewis y antigen in OCCC and normal ovarian tissue

### Expression of ANXA4, Lewis y antigen, and its synthetic key enzyme FucT1, FucT2 in different subtypes of ovarian cancer cell lines

**Figure 3:**
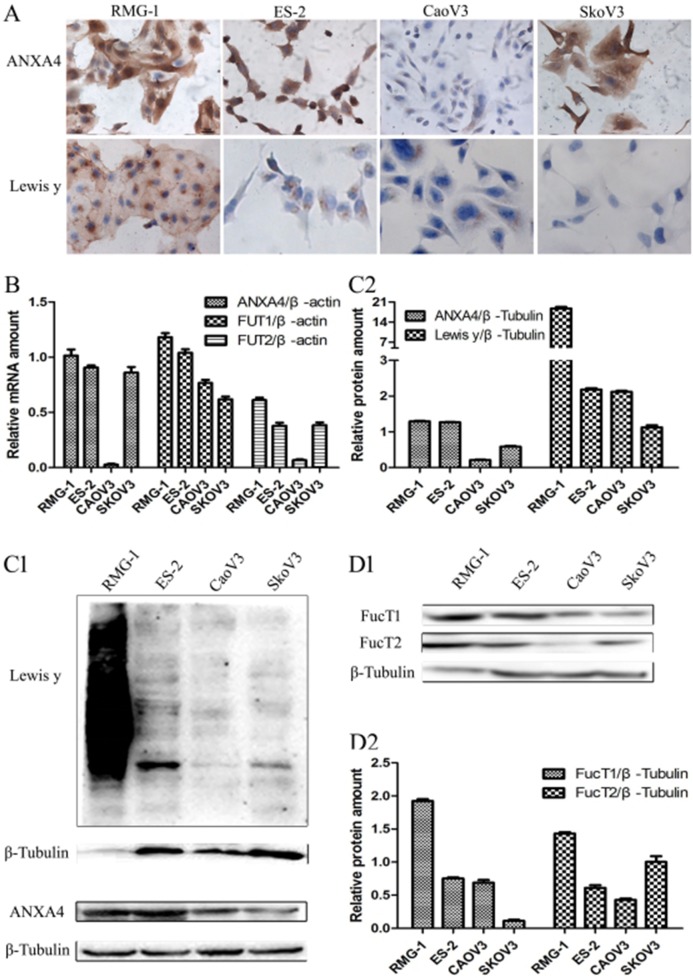
Expression of ANXA4 and Lewis y antigen in OCCC cells (RMG-1, ES-2) and serous ovarian cancer cells (CaoV3, SkoV3) **(A)** ICC detection of ANXA4 and Lewis y antigen expression in four cells; **(B)** real-time PCR detection of ANXA4 mRNA, FUT1 mRNA, and FUT2 mRNA in four cells; **(C)** Western blot detection of ANXA4 and Lewis y antigen expression in four cells; **(D)** Western blot detection of FucT1 and FucT2 expression in four cells.

Both ICC and Western blotting showed significantly greater expression of ANXA4 and Lewis y antigen in the two OCCC cell lines RMG-1 and ES-2 than in the serous OC cell lines CaoV3 and SkoV3 (Figure 3A, 3C1 and 3C2). Expression of the two key enzymes involved in Lewis y antigen synthesis, FucT1 and FucT2, detected using Western blotting, was also found to be significantly higher in RMG-1 and ES-2 cells than in CaoV3 and SkoV3 cells (Figure 3D1 and 3D2). Similarly, significantly greater levels of ANXA4 mRNA and FUT1 mRNA, detected using real-time PCR, were found in RMG-1 and ES-2 cells than in CaoV3 and SkoV3 cells, and FUT2 mRNA and FucT2 protein have different expression on these cells (Figure 3B).

### Relationship between expression of ANXA4 and Lewis y antigen expression, and OCCC clinicopathological parameters

For samples from cancers at later FIGO stages (III/IV), Lewis y antigen had a strong positive expression rate of 85.2%(23/27), significantly higher than that of samples from cancers at the earlier stages (I/II stage), which had a weaker positive expression rate of 54.2%(30/59), *P*<0.01(*P*=0.006). In samples from the chemotherapy resistance group, the strong Lewis y antigen positive expression rate was 89.5% (17/19), significantly higher than 57.7%(30/52) observed in the chemotherapy sensitivity group, *P*<0.01(*P*=0.007). In contrast, in the lymph node metastasis group, the strong positive expression rate of Lewis y antigen was 90.0%(9/10), which was higher than in the group without lymph node metastasis (60.8%,45/74), but not statistically significant, P>0.05(P=0. 145).

The expression of ANXA4 was similar to that of Lewis y antigen; in samples from cancers at later FIGO stages (III/IV), the strong ANXA4 positive expression rate was 81.5% (22/27), significantly higher than45.8% (27/59) found in samples from earlier FIGO stages (I/II), *P*<0.05 (*P*=0.015). The strong ANXA4 positive expression rate in the chemotherapy resistance group was 94.7%(18/19), significantly higher than in the chemotherapy-sensitive group(57.7%, 30/52), *P* <0.05(*P*=0.003). The strong ANXA4 expression rate in the lymph node metastasis group was 90.0%(9/10), which was higher than the group without lymph node metastasis(58.1%, 43/74), but not significant, (*P*<0.05, *P*=0.109). (See Table 2)

**Table 2:** The relationship between the expression of ANXA4 and Lewis y antigen, and OCCC clinicopathological parameters

Characteristics	Cases	ANXA4		p-value	Lewis y		p-value
		Low	High		Low	High	
**Surgical stage**				0.015			0.006
I~II	59	27	32		27	32	
III~IV	27	5	22		4	23	
Lymph node metastasis^*^				0.109			0.145
yes	10	1	9		1	9	
no	74	31	43		29	45	
**Chemosensitivity**				0.003			0.007
sensitive	52	22	30		20	32	
resistant	19	1	18		2	17	

### Relationship between expression of ANXA4 and Lewis y antigen, and OCCC prognosis

COX prognostic multi-factor analysis was carried out for clinical stages, lymph node metastasis, residual tumor sizes, ANXA4 expression levels, and Lewis y antigen expression levels. Clinical stages, ANXA4 expression levels, and Lewis y antigen expression levels were found to be independent risk factors in OCCC prognosis. (Table 3)

**Table 3:** Ovarian clear cell carcinoma prognostic multi-factor analysis

Variables	p-value	Hazard radio (95% CI)
**ANXA4** (low *vs.* high)	0.023	5.463 (1.270-23.497)
**Lewis y** (low *vs.* high)	0.036	4.747 (1.107-20.360)
**Surgical stage** (I-II *vs.* III-IV)	0.004	3.719 (1.523-9.078)

### Survival analysis

**Figure 4:**
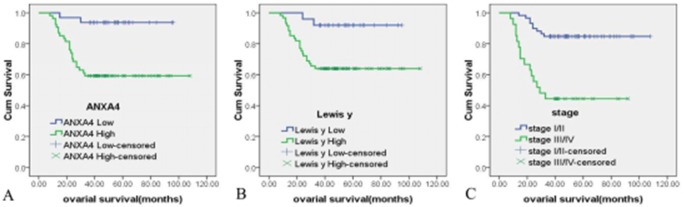
The relationship between ANXA4, Lewis y antigen expression and OCCC patient survival time Kaplan-Meier survival analysis showed that high expressions of ANXA4 **(A)** Lewis y **(B)** and late surgical stage **(C)** were independent risk factors for overall survival. Log Rank *P* = 0.006, 0.0044,0.000.

Kaplan-Meier analysis of patient survival rates versus ANXA4 intensity (Figure 4A) found that the survival rate of patients with high ANXA4 content was lower than that of patients with low ANXA4 content, at each time point, with log rank testing at *P*<0.01(Log Rank *P*=0.001). Kaplan-Meier analysis of patient survival rates versus Lewis y antigen intensity (Figure 4B) found that the survival rate of patients with higher Lewis y antigen expression was lower than of patients with lower expression, at each point, with log rank testing at *P*<0.01(Log Rank *P*=0.009). According to Kaplan-Meier analysis of the survival rate of two groups of patients in various clinical stages (Figure 4C), the survival rate of patients at FIGO III/IV stages was lower than that of patients at FIGO I/II stages, at all time points, with log rank testing at *P*<0.01(Log Rank *P*=0.000).

### Correlation between the expression of ANXA4 and Lewis y

There were 2, 1, 0 and 83 cases in the ANXA4-/Lewis y-, ANXA4+/Lewis y-, ANXA4-/Lewis y+ and ANXA4+/Lewis y+, respectively. Correlation analysis showed that there was a positive correlation between the expression of ANXA4 and Lewis y in OCCC (Spearman correlation coefficient Rs=0.812, *P* < 0.01)(Table 4).

**Table 4:** The correlation between ANXA4 and Lewis y expression in occc

Lewis y	Cases	ANXA4	
		Negative	Positive
**Negative**	3	2	1
**Positive**	83	0	83
**Cases**	86	2	84

### Interaction between ANXA4 and NF-kB p50 in ovarian clear cell carcinoma

**Figure 5:**
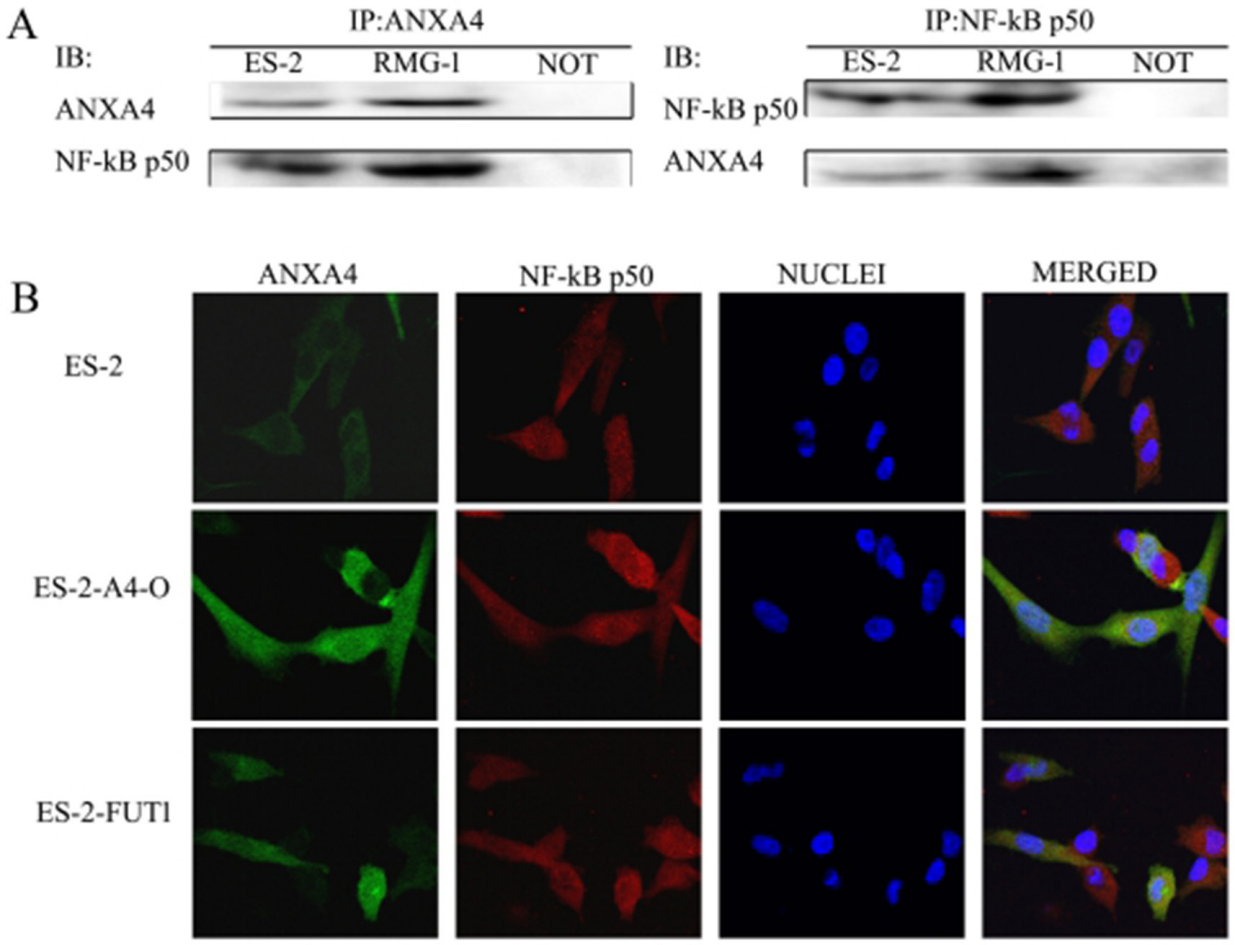
Interaction of ANXA4 and NF-kB p50 **(A)** Cell lysate from ES-2 and RMG-1 cell lines was immunoprecipitated with anti-ANXA4antibody and anti-NF-kB p50 antibody, and then immunoblotted with anti-NF-kB p50 antibody and anti-ANXA4 antibody. **(B)** Double-labeling immunofluorescence showed the colocalization of ANXA4 and NF-kB p50.

Immunoprecipitation was used to detect the expression of ANXA4 and NF-kB p50 in two OCCC cell lines (RMG-1 and ES-2), and an interaction between ANXA4 and NF-kB p50 was found in both RMG-1 and ES-2 cells (Figure 5A).

Green fluorescence-labeled ANXA4 was found to be widely distributed in cytoplasm, nuclei, and cell membranes in both cell types, whereas red fluorescence-labeled NF-kB p50 was found mainly in the cell nuclei and cytoplasm, and blue fluorescence was observed in cell nuclei after DAPI staining. After software analysis of the photographs, yellow fluorescence, due to overlap of green fluorescence-labeled ANXA4 and red fluorescence-labeled NF-kB p50, was observed; indicating co-localization of ANXA4 and NF-kB p50 (Figure 5B). In ES-2-A4-O and ES-2-FUT1 cells, which have high transfection of ANXA4 or FUT1, the fluorescence of both ANXA4 and NF-kB p50 in nuclei was significantly stronger than in ES-2 cells, indicating that expression of both ANXA4 and NF-kB p50 increased after transfection of ANXA4 or FUT1; similarly, the yellow fluorescence indicative of co-localization was also enhanced (Figure 5B).

### ANXA4 fucose glycosylation promotes tumour progression of OCCC by enhancing interactions between ANXA4 and NF-kB p50

**Figure 6:**
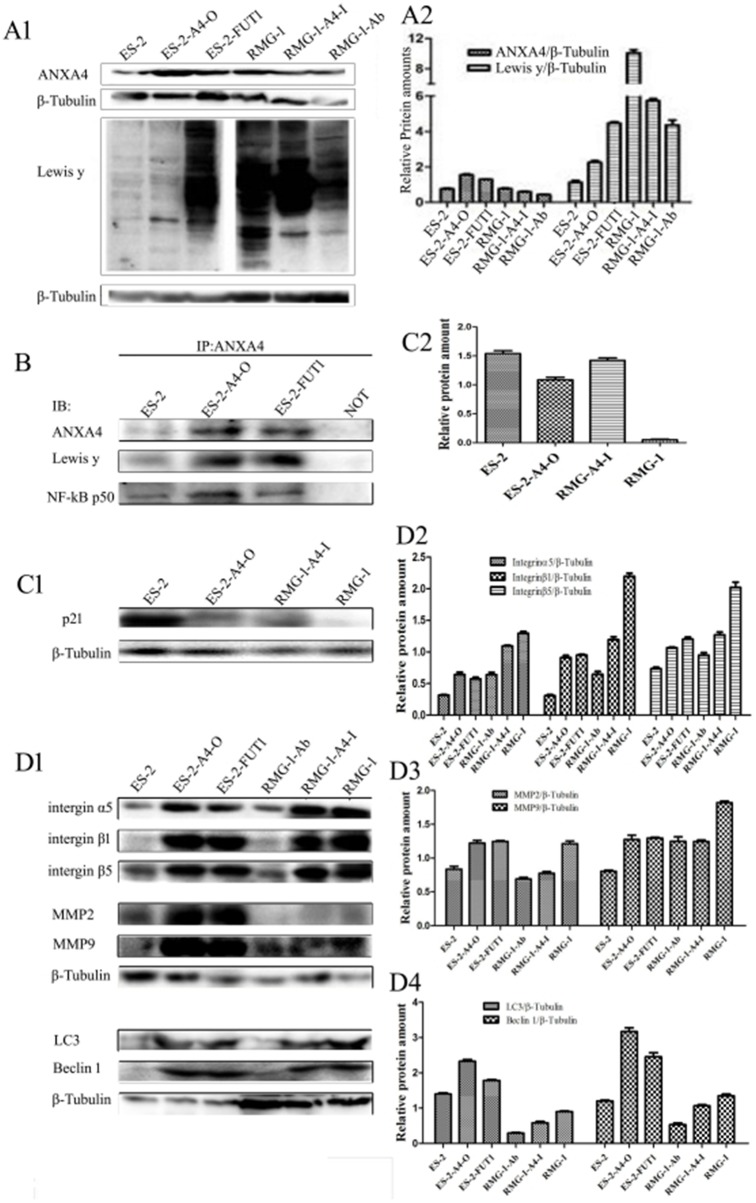
Lewis y antigen modification enhances interactions between ANXA4 and NF-kB p50 **(A)** Expression of Lewis y antigen, ANXA4, and NF-kB p50 in cells before and after treatment; **(B)** Expression of Lewis y antigen and NF-kB p50 on ANXA4 before and after transfection, detected by IP; **(C)** Expression of p21 in cells before and after transfection; **(D)** Changes in expression of integrin α5, β1, β5, MMP2, MMP9, LC3, and Bcl-1 before and after treatment, detected by WB.

**Figure 7:**
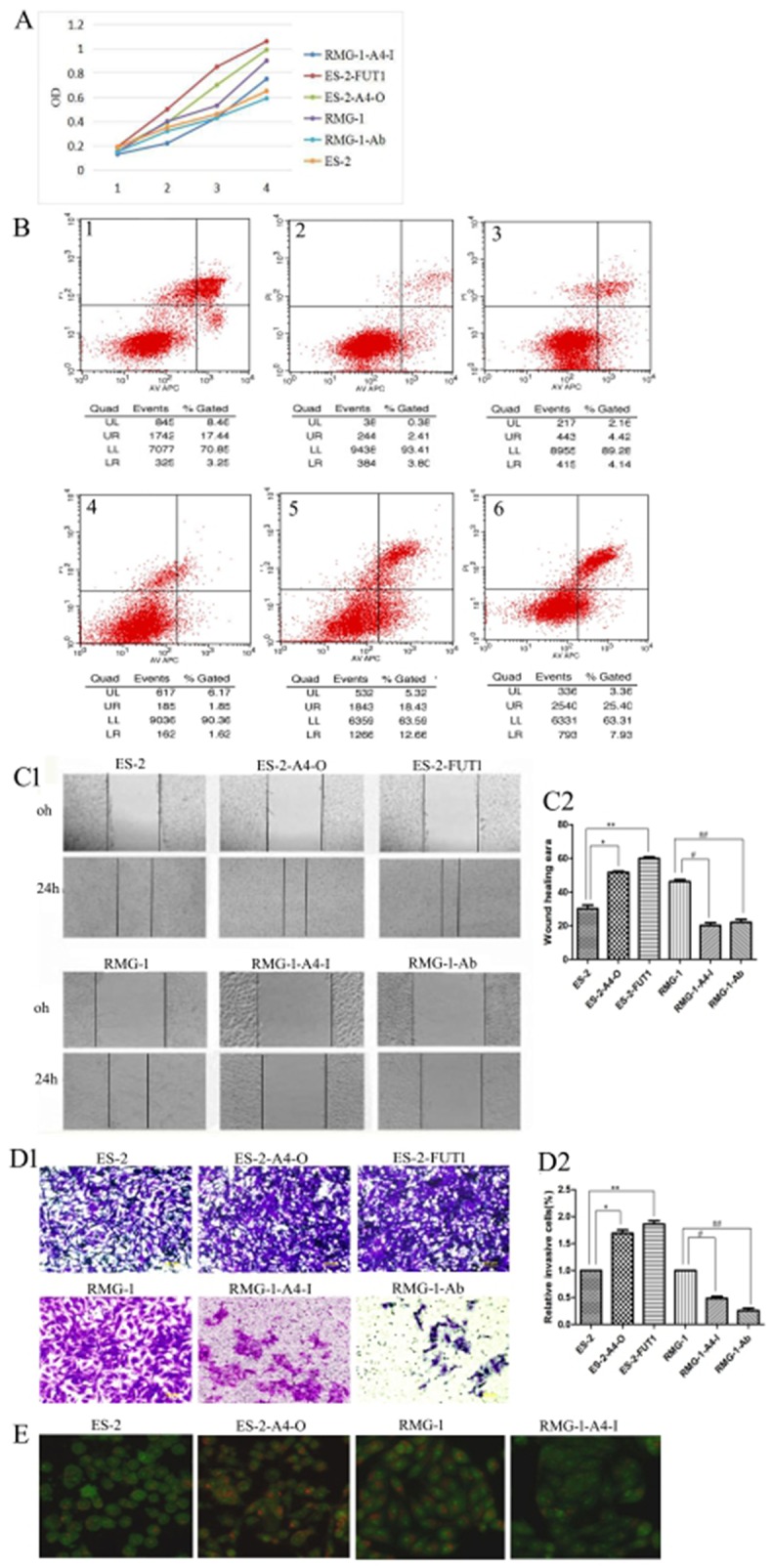
Lewis y modification enhances the role of ANXA4 on promoting OCCC cell proliferation, adhesion, migration, invasion, and autophagy but inhibits cell apoptosis **(A)** Changes in cell proliferation rate before and after treatment, detected by MTT; **(B)** Apoptosis changes before and after treatment detected by APC /PI double staining of annexin-IV (1, ES-2; 2, ES-2-A4-O; 3, ES-2-FUT1; 4, RMG-1; 5, RMG-1-A4-I; 6, RMG -1-Ab); **(C)** Changes in cell migration ability before and after treatment, detected by scratch assay; **(D)** changes in cell invasive abilities before and after treatment, detected by transwell migration assay; **(E)** Intracellular acidic vesicles number and staining intensity, observed by AO staining.

The following cell lines were tested: RMG-1-Ab (Lewis y antigen monoclonal antibodies added to RMG-1 cells), RMG-1-A4-I (*ANXA4* knockdown RMG-1 cells) ES-2-FUTI (ES-2 cells transfected with *FUTI*), and ES-2-A4-O (ES-2 cells transfected with *ANXA4* gene). Western blotting showed that expression of Lewis y antigen, ANXA4, NF-kB p50, integrin α5, β1, β5, MMP2, MMP9, LC3 and Bcl-1 was lower in both RMG-1-Ab and RMG-1-A4-I cells than in RMG-1 cells (Figure 6A and 6D), and p21 expression was enhanced (Figure 6C). In contrast, in ES-2-FUT1 and ES-2-A4-O cells, these same parameters were increased relative to none-transfect ES-2 cells (Figure 6A and 6D), and p21 expression was reduced (Figure 6C). Co-immunoprecipitation assays showed the presence of more Lewis y antigen and NF-kB p50 on ANXA4 in ES-2-FUT1 and ES-2-A4-O cells than in ES-2 cells (Figure 6B). Measures of cell proliferation, adhesion, invasion and migration abilities, numbers of intracellular acidic vesicles and staining intensity in RMG1-Ab and RMG-1-A4-I cells were lower than those in RMG-1 cells, and the apoptosis rate increased (*P*< 0.05). In contrast, measure of cell proliferation, adhesion, migration and invasion abilities and autophagy were higher in ES-2-A4-O and ES-2-FUT1 cells than ES-2 cells, and the apoptosis rate decreased (*P* all <0.05) (Figure 7A, 7B, 7C, 7D and 7E)

## DISCUSSION

During the process of malignant change in cells, the sugar chains that cover the cell change significantly, including changes in their amount and their structure. Lewis y antigen is a terminal oligosaccharide of certain glycoproteins and glycolipids, and is associated with a various processes in tumor cells involved in progression to malignancy [6–10]. Many OC labeled molecules, such as CA125, CD44, and HE4, contain Lewis y antigen structure [8, 11], and Lewis y antigen modifies can enhanced the biological behaviors of tumor cells regulated by these proteins. ANXA4, a member of annexin family, is involved in regulating cell membrane construction, membrane material transport, regulation of ion membrane transport, cell differentiation, and other important biological functions [12]. In the annexin family, we found the presence of Lewis y antigen structure on ANXA2, and that Lewis y antigen promotes ANXA2-mediated tumor proliferation, adhesion and metastasis [13]. Using immunoprecipitation and immunofluorescence double labeling protocols, for the first time, we demonstrates clearly that Lewis y antigen is a part of ANXA4 structure, and that co-localization exists between the two molecules. Then immunohistochemistry shows that the expression of ANXA4 in OCCC tissues is consistent with that of Lewis y antigen, and shows significantly positive correlation between the expression of them (Spearman *r*_s_=0.812).

For futher investigate the expression of ANXA4 and Lewis y, in this study, we used immunohistochemistry and found that the positive expression rate of ANXA4 in OCCC was 97.7%, which was significantly higher than that found in tissues of other subtypes of OC (40.0%) and normal ovarian tissue (0%), all P<0.01. Furthermore, in OCCC cell lines RMG-1 and ES-2, both the expression of ANXA4 mRNA or of ANXA4 protein was significantly higher than in serous OC cell lines, suggesting that ANXA4 may serve as a marker molecule for OCCC. Studies suggest that the expression of ANXA4 in many tumors is specific to particular pathology types and may therefore be a marker molecule for different subtypes of cancers. In a cDNA microarray's study of the expression of ANXA4 in OC, ANXA4 expression was more significantly increased in OCCC compared to serous OC [14], and our results also certified that the expression of ANXA4 in OC is pathologically specific. Our results also suggest that ANXA4 has a higher expression rate in endometrial and cervical clear cell carcinoma than in other types of pathology (results to be published). In renal carcinoma, the positive expression rate of ANXA4 in renal clear cell carcinoma is as high as 87.4%, significantly higher than for other types of pathology [15]. The reason for high expression of ANXA4 in clear cells is still unclear, but studies in the literature suggest that in OCCC, wild-type p53 can combined with the ANXA4 gene upstream transcriptional starting point, expression of ANXA4 in cells is upregulated, but mutant p53 does not produce this effect [16]. It is well known that, wild-type p53 is the most effective tumor suppressor gene, then, after promotion of increased expression of ANXA4 by wild-type 53 has occurred, what role does ANXA4 play in promoting tumor progression in OCCC? Research shows that wild-type p53 can combine with the promoter region of proto-oncogene murine double minute chromosomes 2 (MDM2) to enhance MDM2 protein expression. The resulting increased expression of MDM2 can degrade wild-type p53 protein or inhibit wild-type p53 transcriptional activation by proteolysis, providing negative feedback and regulation of wild-type p53 expression, then promotes tumor growth [17]. It can therefore be hypothesized that, similar to the mode by which MDM2 functions, wild-type p53 promotes high expression of ANXA4 in OCCC. This enhanced expression of ANXA4 then influences p53-mediated transactivation so that p53 function is weakened or lost, leading to progression of OCCC. To test these ideas, we examined changes of p21 protein which is the downstream of p53 in OCCC cells that before and after high transfection or knockdown ANXA4 gene. In the high transfection cells, ANXA4 expression increased, and expression of P21 was reduced compared to the unmodified cells. These results are consistent with the suggestion that increased expression of ANXA4 sends negative feedback to regulate the expression of wild-type p53, and in turn, leads to reduced expression of p21.

Studies have shown that Lewis y antigen is expressed in OC tissues and that increased expression indicates a poor prognosis [10, 18]. Our study examined the expression of Lewis y antigen in normal ovarian tissues and different subtypes of OC tissues, and found that the pattern of Lewis y antigen expression was similar to that of ANXA4 expression. Expression of Lewis y antigen was significantly higher in OCCC tissues (96.5%) than in normal ovarian tissues(0%) and in tissues exhibiting other subtypes of OC (85.5%, all P<0.05). Lewis y antigen and its key synthesis enzyme expression were significantly higher in OCCC cell lines than in serous ovarian carcinoma cell lines. The increased expression of Lewis y antigen in OCCC can be explained due to the increased expression of its synthesis key enzyme α1,2-FUT regulated by α1,2-FUT gene.

Previous studies have shown that, in Hela cells, increases in Ca2+ concentration promotes binding of ANXA4 and NF-kB p50, and then the resulting complex moves from the cell cytoplasm into the nucleus, inhibiting cell apoptosis [19]. Our findings using co-immunoprecipitation and immunofluorescence suggest that ANXA4 also interacts with NF-kB p50 in OCCC. In the high transfection ANXA4 cells, expression of NF-kB p50 increased with ANXA4 expression; expression of the NF-kB p50 downstream molecules integrin α5, β1, β5, MMP2, MMP9, LC3, and Bcl-1 also increased; cell proliferation rate, adhesion, invasion and migration ability, and autophagy increased; but apoptosis decreased relative to the unmodified cells. The results detected in ANXA4 knockdown cells were opposite. These changes suggest that with the increasing expression of both ANXA4 and NF-kB p50, ANXA4/NF-kB complex translocated to nuclei increased, the related DNA sequence motifs bind together to induce an increase in the transcription of target genes, which promotes cell malignancy progression. Previous studies have shown that NF-kB inhibits p53-dependent gene transcription, and ANXA4 can bind NF-kB p50. It was therefore proposed that in OCCC, wild-type p53 activates *ANXA4* transcription, thereby increasing the rate at which ANXA4 is expressed and then combines with NF-kB p50, which in turn facilitates expression of NF-kB downstream related molecules and inhibits p53-dependent gene transcription, which thereby promotes progression to malignancy [20, 21].

Cai's study also showed that ANXA4 is over-expressed and its phosphorylation modification is significantly increased in colon carcinoma tissues relative to normal colon tissues; and that ANXA4 expression is correlated with the late stages of colon cancer and poor prognosis [22]. So, in OCCC, does the observation that both ANXA4 and NF-kB p50 promote tumor progression have anything to do with their modifications? Our previous studies have shown that a variety of tumor-associated glycoproteins have Lewis y antigen structure, such as epidermal growth factor receptor EGFR [23], CD44 [12] and integrin α5β1 [18], and Lewis y antigen modifies the end of the protein, which then affects the biological functions mediated by these molecules, promoting the occurrence and development of tumors. This study examined Lewis y antigen is a part of annexin A4 structure, and high expression of ANXA4 and Lewis y antigen is associated with OCCC FIGO stages and with chemotherapy resistance, with high expression of both being an independent risk factor for OCCC prognosis. To further explore the impact of Lewis y antigen modification on biological behaviors of OCCC, which are regulated by ANXA4, this study used two OCCC cell lines, RMG-1 and ES-2, to carry out Lewis y monoclonal antibodies treatment or *FUT1* gene stable transfection, and detects changes in expression of Lewis y antigen, ANXA4, NF-kB p50, and their downstream related molecules, as well as changes in the biological behaviors of malignant cells before and after treatment. The results make it clear that, after addition of Lewis y monoclonal antibodies to RMG-1 cells, expression of ANXA4 and NF-kB p50, as well as integrin α5β1, β5, MMP2, MMP9, LC3, and Bcl-1 was decreased; cell proliferation, adhesion, invasion and metastasis abilities were decreased; and apoptosis increased. In contrast after transfection of ES-2 with *FUT1* gene, the results were opposite. Therefore, we suggest that modifying ANXA4 by adding Lewis^y^ antigen to its structure enhances the binding of ANXA4 and NF-KB p50, which in turn increases the NF-KB-regulated gene transcriptional activation that influences the proliferation, adhesion, invasion and metastasis, autophagy, and other biological behaviors of OCCC, and therefore ultimately leads to a poor prognosis.

This study demonstrates that significantly high levels of ANXA4 and Lewis^y^ antigen expression occur in OCCC, and shows that Lewis y antigen is a part of the structure of ANXA4. The modifications of ANXA4 by Lewis y antigen affect the binding of ANXA4 and NF-KB p50, and promote tumor progression of OCCC, resulting in a poor prognosis. Therefore, further study of how Lewis y antigen modifications influence the biological functions and mechanisms of ANXA4 will help clarify the ways that ANXA4 promotes tumors and deepen the understanding of the pathogenesis of OCCC and of its malignant biological processes. This may contribute to earlier detection of OCCC, more targeted treatment, and improvements in prognosis.

## MATERIALS AND METHODS

### Specimen sources

Paraffin specimens of surgical resection from 141 cases of ovarian cancer (86 of OCCC, 30 of serous OC, 17 of mucinous OC, 8 of intrauterine membrane-like OC) and 10 normal ovarian tissue paraffin specimens (normal ovarian tissues were from ovariectomized patients with cervical cancer), all of which were from the Department of Obstetrics and Gynecology, Shengjing Hospital of China Medical University (Shenyang, China), from 2003 to 2012, were used in this study. All ovarian cancer patients have not underwent chemotherapy before the surgery, and pathology sections were confirmed by experts after hematoxylin and eosin (HE) staining. The age range of the ovarian clear cell carcinoma group was 29 to 73 years; median age was 50.3 years. The age range of the other EOC subtypes group was 16 to 77 years; median age was 52.8 years. There was no statistically significant age difference among OCCC, other subtypes of ovarian cancer, and normal ovarian patients. The classification of cancer stage was according to the International Federation of Gynecology and Obstetrics (FIGO, 2009). Among the 86 patients with OCCC, 51 were FIGO stage I, 8 cases were FIGO stage II, 23 were FIGO stage III, and 4 were FIGO stage IV. Among these 86 cases, 4 cases refused chemotherapy, 11 patients were lost to follow-up, and other 71 patients were given 6-8 cycles of paclitaxel plus platinum systemic chemotherapy after surgery according to National Comprehensive Cancer Network (NCCN) guidelines and have, so far accepted regular follow-ups; there were 52 cases of sensitivity to chemotherapy and 19 cases of chemotherapy resistance. The outcomes for the 71 patients included 25 deceased patients, 1 patient who survived with recurrence, and 45 patients who survived without tumors. The National Comprehensive Cancer Network (NCCN) criteria states that (1) the chemotherapy-resistant group includes patients who had a clinical response to the initial TC chemotherapy program but experienced subsequent relapse, either in the late stage of chemotherapy or within 6 months after the completion of chemotherapy, and (2) the chemotherapy-sensitive group includes patients who maintained a clinical response for ≥12 months.

### Methods

#### Co-immunoprecipitation

RMG-1 and ES-2 cells derived from an OCCC cell line were plated in RPMI-1640 medium or Macoy 5A with 10% of fetal bovine serum albumin medium, respectively, and incubated in an incubator at 37 °C under 5% CO_2_.

Rabbit anti-human ANXA4 antibodies and rabbit anti-human and mouse anti-human NF-kB p50 antibodies were purchased from Santa Cruz Biotechnology, (Santa Cruz, CA, USA), and mouse anti-human ANXA4 antibodies and mouse anti-human Lewis y antigen antibodies were purchased from Abcam (Cambridge, UK). Details of the experimental procedure have been published elsewhere [8]. Three co-immunoprecipitations were performed: (1) ANXA4 protein was precipitated with rabbit anti-human ANXA4 antibodies and detected using mouse anti-human ANXA4 antibodies, and Lewis y antigen detected using mouse anti-human Lewis y antigen antibodies; (2) ANXA4 protein was precipitated with rabbit anti-human ANXA4 antibodies and detected with mouse anti-human ANXA4 antibodies, and NF-kB p50 was detected with mouse anti-human NF-kB p50 antibodies: and (3) NF-kB p50 was precipitated with rabbit anti-human NF-kB p50 antibodies and detected with mouse anti-human NF-kB p50 antibodies, and ANXA4 protein was detected using mouse anti-human ANXA4 antibodies. The primary antibody was replaced by rabbit IgG (Bioss, China) as negative control. An enhanced chemoluminescence reagent was used (Amersham ECL Prime Western Blotting Detection Reagent; GE Healthcare Life Sciences, Little Chalfont, UK), and the experiment was repeated three times.

#### Confocal laser scanning microscopy

Two immunofluorescence double labeling experiments were carried out. For both, protocols strictly followed the instructions of the reagent suppliers. (1) Rabbit anti-human ANXA4 antibodies (1:50, Santa Cruz) and mouse anti-human Lewis y antigen antibodies (1:50, Abcam) were simultaneously added to monolayered cell slides prepared from ES-2 cells. To these, the following secondary antibodies were applied: fluorescein isothiocyanate (FITC) green fluorescence-labeled mouse IgM fluorescence (1:8) and tetramethylrhodamine (TRITC) red fluorescence-labeled rabbit IgG (1:50). Cell nuclei were stained with 4’,6-diamidino-2-phenylindole (DAPI). (2) Monolayered cell slides were prepared from ES-2 cells, high-efficiency ANXA4 ES-2-A4-O cells and high-efficiency FUT1 ES-2-FUT1 cells. Mouse anti-human ANXA4 antibodies (1:100, Abcam) and rabbit anti-human NF-kB p50 antibodies (1:50, Santa Cruz) were added to cell slides. Secondary antibodies were FITC green fluorescence-labeled mouse IgG (1:50), and TRITC red fluorescence-labeled rabbit IgG (1:50); cell nuclei were stained with DAPI. For negative controls, phosphate buffered saline (PBS) replaced primary antibodies. Double-labeled immunofluorescence samples were viewed by fluorescence confocal microscopy.

#### Immunohistochemistry

Expression of ANXA4 and Lewis y antigen was detected using streptavidin-peroxidase (SP) and Strept Avidin-Biotin Complex (SABC) immunohistochemistry staining, using rabbit anti-human ANXA4 antibodies (1: 200, Abcam) and mouse anti-human Lewis y antigen antibodies (1:700, Abcam). All other reagents were provided by the central laboratory of Shengjing Hospital of China Medical University (Shenyang, China). Each experimental trial included OCCC, serous OC, mucinous OC, endometrial-like OC and normal ovarian tissue sections (see 1.2.1 Specimen sources for the proportions of the cell types), and as well as both positive and negative control sections. The positive control sections for ANXA4 were renal clear cell carcinoma tissues, and for Lewis y antigen, were gastric carcinoma tissues. For negative controls, PBS was substituted for primary antibodies. Staining protocols were pursuant to the manufacturer's instructions for the reagent kit.

Immunohistochemistry results were interpreted as previously described [24]. Samples were marked positive when the cell membrane or cytoplasm appeared brown or yellow. The 86 OCCC samples were separated into high expression (++ / +++) and low expression (− / +) groups according to levels of ANXA4 and Lewis y antigen staining. Each sample was independently assessed by two people, and again by a third person when inconsistencies arose.

#### Immunocytochemistry

Monolayer cell slides were prepared from four kinds of ovarian cancer cells, RMG-1, ES-2, CaoV3, and SkoV3, while they were in the exponential growth phase. Reagents were as described for the immunohistochemistry assay; antibodies were rabbit anti-human ANXA4 (1:100, Santa Cruz) and mouse anti-human Lewis y antigen antibodies (1:100, Abcam). All procedures were as described in reagent kit instructions.

#### Western blotting

Western blots were prepared as previously described [8]. Antibodies used were: rabbit anti-human ANXA4 (1:200, Santa Cruz), mouse anti-human ANXA4 (1:1000, Abcam), mouse anti-human Lewis y antigen(1:500, Abcam), rabbit anti-human NF- KB p50 (1:200, Santa Cruz), mouse anti-human NF-kB p50 (1:200, Santa Cruz), rabbit anti-human integrin α5 (1:500, Proteintech, Wuhan, China), rabbit anti-human integrin β1 (1:500, Proteintech), rabbit anti-human integrin β5 (1:200, Beijing Biosynthesis Biotechnology Co., Ltd., Beijing, China), rabbit anti-human MMP2 (1:500, Proteintech), rabbit anti-human MMP9 (1:500, Proteintech), rabbit anti-human LC-3 (1:1000, Abcam), and rabbit anti-human Bcl-1 (1:1000, Cell Signaling Technology, Danvers, MA, USA).

#### Quantitative real-time PCR

RNA was extracted from pretreated cells with RNAiso Plus reagent (TAKARA) and reverse-transcribed by using a Prime-Script RT reagent Kit (TAKARA). Reaction conditions were 37°C for15 min, 85°C for 5 s, 4°C for 5 min. Real-time PCR and Ct value analysis were performed with a Roche LightCycler system. Primers for ANXA4: forward primer: 5′-AGCCTACAAGAGCACCATCG-3′, reverse primer: 5′-GACAGAGACACC AGCACTCG-3′; FUT1: forward primer: 5′-CATGTGGCTCCGGAGCCATCGTC-3′, reverse primer: 5′-GCTCTCAAGGCTTAGCCAATGTCC-3′; FUT2: forward primer: 5′-ATCATGACCATTGGGACGTT-3′, reverse primer: 5′-GTGCTTGAGTAAGGGGGA CA-3′; GAPDH: forward primer: 5′-CCTTCATTGACCTCCACTAC-3′, reverse primer: 5′-GTTGTCATACTTCTCATGGTTC-3′. The real-time PCR reaction conditions were denature at 95 °C for 30°C 40 cycles of 95°C for 5 s and 60°C for 30 s in a 20-ul reaction mixture containing SYBR^@^ Premix Ex Taq™(2×) 10 μL, PCR Forward Primer(10 μmol/L)0.4 μL, PCR Reverse Primer(10 μmol/L)0.4 μL, cDNA 2 μL, dH2O 7.2μL. GAPDH was used as the endogenous control. Once the amplification was completed, the melting curve was analyzed. The change of target gene expression level was calculated using the 2^-ΔΔCT^ method.

#### Gene transfection

Transfection reagent kits were purchased from (Invintrogen Co., Carlsbad, CA, USA), and the kit protocol was followed for all transfections. The following stable cell lines were generated: ES-2-FUT1, E2-2 cells transfected with Pc-FUT1-GFP plasmids (Takara Bio Inc., Katsatsu, Japan); ES-2-A4-O, ES-2 cells transfected with pc-ANXA4-GFP plasmids (Zimmer AG); and RMG-1-A4-I, RMG-1 cells transfected with Sh-ANXA4-GFP plasmids (Zimmer AG).

#### Thiazolyl blue tetrazolium bromide (MTT) assay

Six cell lines were assayed: ES-2, ES-2-FUT1, ES-2-A4-O, RMG-1, RMG-1-A4-I, and RMG-1-Ab; RMG-1-Ab denotes cells to which Lewis y antigen antibodies (10 ug/ml) were added when RMG-1 cells were loaded. The 0 point was after six hours of cell culture; optical density (OD) was measured at the 0 point and then after 24, 48, and 72 hours. The experiment was repeated three times.

#### Cell apoptosis

Cell apoptosis was detected using flow cytometry in the cell lines ES-2, ES-2-FUT1, ES-2-A4-O, RMG-1, RMG-1-A4-I, and RMG-1-Ab; RMG-1-Ab group cells were generated as described for the MTT assay. These cell lines were divided into two groups, with ES-2, ES-2-FUT1, ES-2-A4-O in one group, and RMG-1, RMG-1-Ab, RMG-1-A4-I in another. Apoptosis in each group was detected by double staining with allophycocyanine (APC) annexin-IV conjugate (annexin-IV-APC) and propidium iodide (PI), using an annexin-IV-APC / PI kit (Nanjing KGI Biotechnology Development Co., Ltd. Nanjing, China), the kit instructions were strictly adhered to. Blanks were run as controls for both PI staining and annexin IV-APC staining for each group. The ratio of cells in early apoptosis and late apoptosis were calculated, and statistical analysis of the data was performed. The experiment was repeated three times.

#### Transwell invasion experiment

The ECM gel was diluted by 1:8 with serum free medium, Matrigel 60 ul was added into the upper chambers, then they were placed in an incubator at 37°C overnight. ES-2, ES-2-FUT1, ES-2-A4-O, RMG-1, RMG-1-A4-I, and RMG-1-Ab cell lines were cultured in zero serum medium. For each cell line, 200 μl was added to the upper chamber of transwells (Corning, Tewksbury, MA, USA), and 500 μl fetal bovine serum (FBS) was added to the lower chamber. The transwells were then cultured for 36 hours, and processed as previously described [15]. Data for statistical analysis was collected by counting five horizon cells under a 100 X microscope lens. The experiment was repeated three times.

#### In vitro scratch assay

Fully grown, plated cells of ES-2, ES-2-FUT1, ES-2-A4-O, RMG-1, RMG-1-A4-I, and RMG-1-Ab were scratched and then photographed at 0 hours and 24 hours, as previously described [15]. The experiment was repeated three times.

#### Acridine orange staining

To detect cell autophagy, ES-2, ES-2-A4-O, RMG-1 and RMG-1-A4-I cells were stained with acridine orange to observe the number of inner acidic vesicles (acidic vesicular organelles, AVOs) and staining intensity.

### Statistical methods

SPSS 17.0 statistical software was applied for statistical analysis, with all data represented by *x* ±*s*. Statistical methods were varied based on data type: the χ^2^ test was used for semi-quantitative data whereas the analysis of variance (ANOVA) test, the χ^2^ test, and the Wilcoxon rank sum test were used for quantitative data.
